# Training-Free Defect Image Generation with Multi-Domain Consistency and Geometric-Semantic Constraints for Industrial Visual Sensing Inspection

**DOI:** 10.3390/s26134216

**Published:** 2026-07-03

**Authors:** Yushen Wang, Dengbiao Jiang, Yiming Wang, Kelong Zhu, Guoquan Yao

**Affiliations:** 1 School of Computer, Jiangsu University of Science and Technology, Zhenjiang 212003, China; 232241807605@stu.just.edu.cn (Y.W.); 231210701133@stu.just.edu.cn (K.Z.); 2 Tofflon Science & Technology Group Co., Ltd., Shanghai 201108, China; yiming@tofflon.com; 3 Key Laboratory of High Performance Ship Technology of Ministry of Education, Wuhan University of Technology, Wuhan 430063, China; yaogq@whut.edu.cn

**Keywords:** industrial defect generation, training-free diffusion model, multi-domain consistency, geometric semantic constraints, anomaly sample augmentation

## Abstract

Industrial defect generation has long been challenged by the scarcity of real anomaly samples and the imbalance of defect categories, particularly in complex industrial scenarios involving transparent containers. Taking vials as an example, glass reflection, specular highlights, and fine-grained defects make continuous defect acquisition difficult, thereby making the realism and controllability of augmented samples critical to downstream detection performance. Although existing diffusion-based generation methods can improve synthetic image quality, they often require additional training or lightweight fine-tuning, which limits their efficiency in sample-limited industrial scenarios. To address this issue, this paper builds upon the TF-IDG framework and proposes a training-free industrial defect generation method based on multi-domain consistency and geometric-semantic constraints. To alleviate the unnatural texture details, boundary transitions, and background blending commonly observed in generated defects, a multi-domain consistency constraint is introduced to enhance generation realism from both frequency-domain structures and cross-domain contextual representations, thereby improving anomaly texture expression and overall visual coherence. To further mitigate unstable defect contours, spatial deviation, and structural mismatch with target objects, a geometric-semantic constraint is designed to regulate the generation process through elastic shape constraints and semantic region-anchored attention, enhancing the rationality of defect morphology evolution and spatial localization. Experimental results on both the MVTec AD dataset and a self-built vial defect dataset demonstrate that the proposed method outperforms comparative approaches. Specifically, when YOLOv11 is used as the downstream detector, the mAP@50 on the MVTec AD dataset and the self-built vial defect dataset is improved from 88.5% and 98.0% for the TF-IDG baseline to 89.6% and 98.8%, respectively.

## 1. Introduction

Industrial anomaly detection is important in pharmaceutical manufacturing, precision electronics, and high-end equipment [1]. Subtle defects on product surfaces or inside products can directly affect performance, stability and operational safety [2]. For data-driven industrial vision methods, model performance depends not only on the detection framework itself but also on whether the training data can sufficiently cover the appearance distribution and structural variation in real anomalies [3,4].

However, anomalous samples are usually scarce in real industrial environments [3]. Normal samples are easier to collect, whereas real defect samples are limited in quantity and often show a clear long-tail distribution across categories. As manufacturing processes improve, low-frequency, occasional, and fine-grained defects become harder to collect continuously, and annotation cost also increases [2]. Under these conditions, detection models tend to favor majority classes and often perform poorly on complex or few-shot defects. Therefore, industrial anomaly sample augmentation has become an important way to improve industrial vision systems.

In the above industrial inspection scenarios, transparent container objects usually pose greater challenges for both generation and detection [5]. Taking vials as an example, their appearance inspection typically relies on an online quality inspection system composed of industrial cameras, directional light sources, clamping and rotation mechanisms, and visual inspection modules. Owing to the pronounced reflection, specular highlights, and edge refraction characteristics of glass materials, defects on vial surfaces, such as cracks, glass spikes, scratches, and stains, often exhibit low contrast, small scale, and locally fine-grained features. Meanwhile, variations across production batches, illumination conditions, and background settings further increase the visual uncertainty of anomalous regions. Therefore, vial inspection is not only a representative application scenario in industrial vision-based sensing and inspection but also provides a challenging and practical testbed for evaluating the realism, background integration capability, and spatial controllability of defect image generation methods.

To alleviate anomalous sample scarcity, researchers have proposed many industrial defect image generation methods. In recent years, diffusion models have become an important technical route because they provide high generation quality and strong fine-detail representation [6,7,8]. However, many diffusion-based methods still require additional training or lightweight fine-tuning, which increases the adaptation cost for new industrial scenarios [9]. Training-free generation provides an alternative by using pretrained generative priors for inference-stage defect transfer and anomaly synthesis. Although existing training-free diffusion methods have been able to generate defect images to a certain extent, their practical applicability in complex industrial scenarios is still limited by insufficient realism and controllability. First, most of these methods mainly rely on local feature alignment and mask-based control in the spatial domain, while providing insufficient constraints on texture frequency structures and background contextual consistency. As a result, the generated images are prone to unnatural boundary transitions, over-smoothed local textures, and inadequate background integration, indicating that their realism remains limited [10]. Second, the controllability of existing methods generally remains at the region level, lacking continuous geometric priors and semantic support-domain constraints. Consequently, they may suffer from unstable defect contours, positional deviations, and mismatches with the target structure, which further limit their controllability [11]. For industrial objects such as transparent containers, where background interference is stronger, textures are more fragmented, and structural constraints are stricter, the above problems become even more prominent.

Therefore, the core research gap in existing training-free industrial defect generation methods lies in how to simultaneously improve the visual realism and spatial controllability of generated defects without introducing additional training costs. On the one hand, generated samples need to be closer to real defects in terms of texture frequency, boundary transitions, and background integration, so as to avoid obvious domain discrepancies between augmented samples and real industrial images. On the other hand, generated defects should also conform to the structural logic of industrial objects in terms of shape evolution and regional placement, thereby ensuring that the augmented data can effectively support the training of downstream detection models. If defect image generation remains limited to the transfer of local anomalous appearances and lacks joint constraints on multi-domain consistency and geometric-semantic rationality, the generated samples may exhibit visually anomalous characteristics, but their practical benefits for industrial vision-based sensing and inspection systems will still be limited.

To address these issues, this paper builds upon the TF-IDG framework [12] and introduces multi-domain consistency and geometric-semantic constraints for industrial defect generation. The multi-domain consistency constraint improves realism from the perspectives of frequency-domain structure and cross-domain context, so that anomalous regions are closer to real industrial defects in texture detail, boundary transition, and background blending. The geometric-semantic constraint improves controllability from the perspectives of shape evolution and regional placement, so that defects not only look anomalous but also appear in reasonable component regions and structural positions. By integrating these two constraints into the inference process, the proposed method improves generation realism, structural rationality, and task adaptability.

The main contributions of this paper are as follows.

1. To address insufficient texture details, unnatural boundary transitions, and poor background blending in existing training-free methods, we construct a multi-domain consistency constraint mechanism. This mechanism improves defect realism from the perspectives of frequency-domain structure and cross-domain context, and enhances the naturalness of anomalous regions in texture expression, boundary transition, and background blending;
2. To address instability, positional deviation, and mismatch with target structures in defect contour evolution and spatial placement, we construct a geometric semantic constraint mechanism. Through elastic shape constraints and semantic region-anchored attention regulation, this mechanism improves the rationality of defect contour evolution and spatial placement, thereby enhancing generation controllability;
3. Experiments on the MVTec AD dataset and the self-constructed vial defect dataset demonstrate that the proposed method can effectively improve the quality of defect image generation and provide more valuable augmented samples for downstream detection tasks in industrial visual sensing systems.

## 2. Related Work

### 2.1. Industrial Defect Generation Methods

To address the shortage of industrial defect samples, researchers have proposed many defect image generation and data augmentation methods. Early work mainly relied on manually designed anomaly synthesis strategies, such as Cut-Paste [13], DRAEM [14], and PRN [15]. These methods usually superimpose anomalous textures onto normal images through cropping, splicing, or local perturbation. They are simple to implement, computationally efficient, and convenient for synchronized annotation, so they were widely used in early industrial anomaly detection studies. However, they still operate at the level of pixel replacement. Although they can create visually salient anomalies, they often fail to preserve the texture continuity, structural consistency, and background harmony required by real defects. As a result, research on industrial defect generation has gradually shifted from manual editing to data-driven methods that aim to learn defect distributions directly rather than paste anomalies with fixed rules.

Against this background, generative adversarial network (GAN)-based methods became an important direction for industrial defect generation. Representative methods include SDGAN [16], DefectGAN [17], DFMGAN [18], and DG2GAN [19]. These methods learn image distributions to generate new anomalous samples. Compared with manual splicing, they can reduce the rigidity of generated results and move defect generation from rule-based construction toward semantic modeling. For example, DFMGAN supports defect generation in few-shot settings through a two-stage strategy, which gives it practical value in small-sample industrial scenarios. However, GANs also have clear limitations. Their training relies on adversarial optimization and is prone to instability, mode collapse, and output fluctuation. These problems become more serious when defect samples are already scarce. To obtain more natural defect textures and more stable generation results, research has gradually shifted to diffusion models.

Diffusion models have been widely used for industrial defect generation because they produce high-quality images and represent fine details well. Relevant methods include DualAnoDiff [20], AnoDiff [21], and AnoAny [22]. These methods usually adapt pretrained diffusion models to industrial scenarios through DreamBooth [23], Textual Inversion [24], and LoRA [25], and use them for anomaly concept learning, defect transfer, and conditional generation. Compared with GANs, diffusion models generally perform better in fine-grained texture representation and overall visual naturalness, and have, therefore, become a mainstream direction in this field. However, high visual generation quality does not necessarily imply stable control over defect textures, shapes, and spatial locations. Although methods such as AnoDiff and AnoAny can generate visually prominent anomalous regions, they lack explicit constraints on the frequency-domain structure of defect textures, making it difficult to stably control fine-grained texture energy, edge sharpness, and local frequency distributions. Meanwhile, their control over defect morphology and spatial position typically remains at the coarse-grained region level, without continuous geometric priors or component-level semantic support-domain constraints. As a result, the generated defects are prone to problems such as unstable contours, positional deviations, or mismatches with the target structure. Therefore, how to generate industrial defect samples that are more realistic, more natural, and more controllable under low-cost conditions remains an important issue to be further addressed in this field. Motivated by this problem, this paper incorporates frequency-domain structure, cross-domain context, and background integration into a unified multi-domain consistency constraint. Furthermore, geometric-semantic priors are introduced through elastic shape constraints and semantic region-anchored attention. In this way, frequency-domain constraints and geometric-semantic priors are integrated into a training-free inference framework, so as to simultaneously improve the realism and controllability of generated defects.

### 2.2. Training-Free Industrial Defect Generation Methods

Training-free industrial defect generation uses the visual priors of pretrained models to synthesize anomalies during inference. These methods usually take a normal image, reference defect samples, and local region constraints as inputs and transfer defect patterns to the target image through image editing, feature alignment, or conditional guidance without updating the generative backbone.

Several representative studies have already shown the potential of this technical route. For example, TF-IDG proposed by Xu et al. in 2025 [12] applies diffusion models to industrial defect generation and establishes a training-free defect generation framework, providing a representative idea for small-sample industrial anomaly data augmentation. RealNet [26] generates anomalous responses by regulating the denoising process without updating model parameters. In addition, example-guided image-editing-based defect synthesis methods show that flexible local defect transfer can be achieved during inference with the help of reference anomalous samples and regional conditions. Together, these studies demonstrate the feasibility of transferring pretrained generative priors to industrial anomaly synthesis tasks within a training-free framework.

From an application perspective, this paradigm reduces the adaptation cost for target industrial scenarios and remains usable under few-shot or one-shot defect conditions. It is, therefore, suitable for anomaly sample augmentation when real defect samples are scarce or when imaging conditions change across products and production batches.

However, current training-free industrial defect generation methods still have several shortcomings. First, they mainly impose constraints on local regions in the spatial domain and pay insufficient attention to frequency structure and deep feature distributions. This often leads to defects with unnatural texture details, boundary transitions, and background blending. Second, existing methods still control defect shape and spatial placement mainly at the region level and lack continuous geometric priors and semantic support-domain constraints. As a result, they are prone to unstable contours, positional deviation, and mismatch with target structures. Finally, although some methods can generate visually obvious anomalies, the results often look like pasted anomalies rather than defects that naturally grow from the image. This weakens the practical value of augmented samples for downstream detection tasks.

Overall, manual editing methods are limited by realism, training-based generative methods are limited by cost and stability, and existing training-free methods still show a clear gap between realism and controllability. In contrast, the improvement proposed in this paper goes beyond local appearance transfer. It extends realism constraints to frequency-domain structure and cross-domain context, so that generated defects are closer to real industrial defects in texture frequency distribution, boundary transition, and background blending. At the same time, instead of keeping control at the level of simple region restriction, this paper further constrains continuous contour evolution and component-level semantic support domains through geometric-prior guidance and semantic region-anchored attention regulation. As a result, the generated defects better match the structural logic of industrial objects in both shape tendency and spatial placement. These improvements further enhance the realism, structural rationality, and task adaptability of industrial defect generation while maintaining the inference-only nature of the original TF-IDG paradigm.

## 3. Method

### 3.1. Overall Framework

Building on the original TF-IDG framework, this paper proposes an industrial defect generation framework based on multi-domain consistency and geometric-semantic constraints. The diffusion backbone remains frozen, and the proposed constraints are introduced during inference to regulate defect generation. These two constraints jointly improve the realism, structural rationality, and spatial controllability of the generated results.

**Figure 1:**
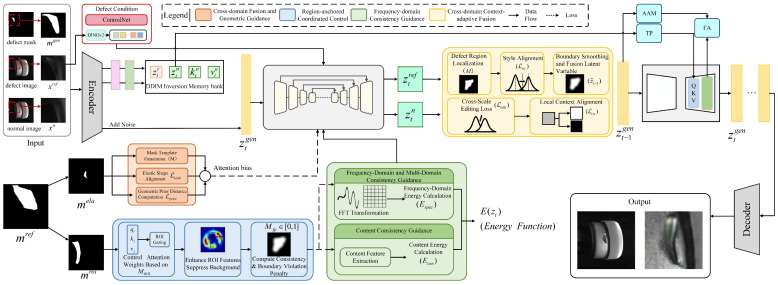
Overall framework of the proposed method.The long dots in the figure indicate omitted intermediate diffusion sampling steps.

The overall pipeline is shown in Figure 1. The inputs include a normal image, a reference defect image, a reference defect mask, and a target generation region. The normal image provides background texture and structural information. The reference defect image and its mask provide anomalous appearance priors. The target generation region constrains the main range of defect generation. In the basic generation branch, the normal image is first mapped to a latent representation by an encoder. The reference defect information is then injected into the diffusion backbone through a reference guidance module to constrain category semantics and local defect appearance. At the same time, the target-region mask is used as a spatial condition to restrict generation location and provide an initial constraint for later structural control. After diffusion sampling and decoding, a defect synthesis result that remains coordinated with the background is obtained.

On this basis, two core constraint mechanisms are introduced to address the two key issues of generation realism and controllability. The multi-domain consistency constraint acts on texture, boundaries, and context, and improves defect realism in terms of frequency structure, local transition, and overall style. The geometric semantic constraint acts on shape evolution and spatial distribution and stabilizes defect contours while constraining the effective support domain of anomalous responses. In this way, generated defects not only look anomalous but also appear in reasonable component regions and structural positions. Both constraints regulate the update direction of latent variables during sampling, which enables collaborative control of defect generation without changing the parameters of the diffusion backbone.

### 3.2. Multi-Domain Consistency Constraint

In industrial defect image generation, realism depends not only on whether defects have a reasonable spatial appearance, but also on whether their texture frequency structure is close to that of real defects and whether they can form natural visual transitions with the target background. Existing training-free methods mainly rely on feature matching and mask control in the spatial domain. Although this is useful for preserving coarse defect semantics, it is not sufficient for modeling fine-grained texture distributions and cross-domain background adaptation. To address this issue, this paper constructs a multi-domain consistency constraint from two aspects: frequency-domain structure and contextual statistics. As a result, generated defects are closer to real defects not only in morphology, but also in local texture and overall visual appearance. This module computes frequency-domain and contextual guidance terms before each DDIM update and uses them to correct the latent variables.

#### 3.2.1. Frequency-Domain Consistency Guidance Mechanism

**Figure 2:**
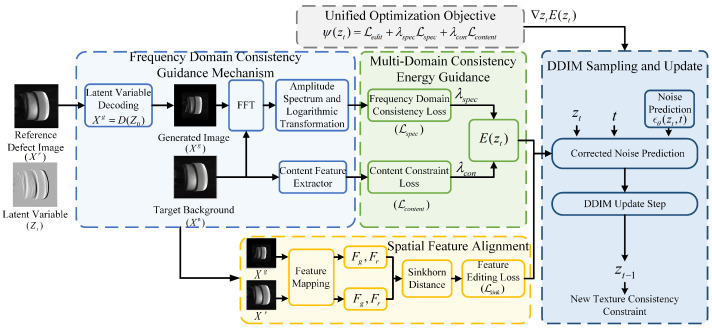
Mechanism of frequency-domain consistency guidance.

Relying only on spatial-domain feature alignment is insufficient to ensure realistic industrial defect generation. Industrial defects often have relatively stable spectral characteristics, and different defect types may differ greatly in medium- and low-frequency structures as well as high-frequency texture details. If the generation process focuses only on local spatial similarity without explicitly constraining frequency composition, the generated result may resemble the reference defect in overall shape but still differ from real defects in local texture intensity, edge sharpness, and detail hierarchy. To address this problem, this paper introduces a frequency-domain consistency guidance mechanism that explicitly constrains the frequency structure of generated anomalies during diffusion sampling. The pipeline of the frequency-domain consistency guidance mechanism is shown in Figure 2. In a diffusion generative model, given the latent variable at the current time step, the denoising network predicts its noise component. According to the DDIM sampling formulation, an estimate of the original latent variable can be obtained [27]:

(1) $${\hat{Z}}_{0}=\frac{Z_{t}-\sqrt{1-{\bar{\alpha }}_{t}}\;\varepsilon_{\theta }\left(Z_{t},t\right)}{\sqrt{{\bar{\alpha }}_{t}}}$$

where $Z_{t}$ denotes the latent variable at the *t*-th diffusion step; ${\hat{Z}}_{0}$ denotes the estimate of the original noise-free latent variable inferred from the current latent variable and $\varepsilon_{\theta }\left(Z_{t},t\right)$ denotes the noise component predicted by the denoising network at time step *t*. The parameter ${\bar{\alpha }}_{t}$ denotes the cumulative retention coefficient up to step t and is usually written as ${\bar{\alpha }}_{t}=\prod_{s=1}^{t}\alpha_{s}$, where $\alpha_{s}=1-\beta_{s}$, and $\beta_{s}$ is the noise schedule parameter in the forward diffusion process.

Then one DDIM update step can be written as

(2) $$Z_{t-1}=\sqrt{{\bar{\alpha }}_{t-1}}{\hat{Z}}_{0}+\sqrt{1-{\bar{\alpha }}_{t-1}-\sigma_{t}^{2}}\;\epsilon_{\theta }\left(Z_{t},t\right)+\sigma_{t}\xi ,\;\xi ∼N\left(0,I\right)$$

where $\sigma_{t}$ is the stochasticity control parameter at time step t, and ξ∼N(0,I) denotes random noise sampled from a standard Gaussian distribution. Traditional DDIM updates are fully determined by noise prediction, and therefore, do not explicitly constrain defect texture structure. To guide sampling toward the distribution of real defects, this paper introduces an energy-guidance term to correct the noise prediction:

(3) $${\tilde{\epsilon }}_{\theta }\left(Z_{t},t\right)=\epsilon_{\theta }\left(Z_{t},t\right)-\eta_{t}\nabla_{Z_{t}}\psi \left(Z_{t}\right)$$

where $\psi (Z_{t})$ is the energy function defined on the current latent variable, $\nabla_{Z_{t}}\psi \left(Z_{t}\right)$ denotes the gradient of this energy with respect to the latent variable, and $\eta_{t}$ is the guidance-strength coefficient. Through Equation (3), the sampling process is guided not only by the noise-prediction ability of the diffusion model, but also by an external structural consistency objective. To construct this energy function, we first consider consistency in spatial-domain feature distributions. Let $\phi_{l}\left(\cdot \right)$ denote the feature map extracted from the l-th layer of the diffusion backbone or an external visual encoder. The features of the current generated image and the reference defect image at layer l are then given by

(4) $$F_{l}^{gen}=\phi_{l}\left(d(Z_{t})\right),\;F_{l}^{r}=\phi_{l}\left(X^{r}\right)$$

where $d(Z_{t})$ denotes the generated image decoded from the current latent variable, $F_{l}^{gen}$ denotes the l-th layer feature of the generated image, and $F_{l}^{r}$ denotes the l-th layer feature of the reference defect image. To align the two feature sets at the distribution level, this paper adopts the entropy-regularized optimal transport distance, namely the Sinkhorn distance, to construct the spatial-domain feature editing loss:

(5) $$L_{sink}=\min_{P\in \prod (\mu ,\nu )}\sum_{i,j}P_{i,j}C_{i,j}-\varepsilon_{s}H\left(P\right)$$

where *P* is the transport matrix, ∏(μ,ν) denotes the set of feasible transports that satisfy marginal constraints, $C_{i,j}$ is the matching cost between generated feature point *i* and reference feature point *j*, $\varepsilon_{s}$ is the entropy regularization coefficient, and H(P) is the entropy of the transport matrix. This term helps the generated defect remain consistent with the reference defect in both coarse semantics and local structure.

However, spatial-domain feature alignment alone still cannot explicitly constrain the frequency composition of textures. Industrial defects often show specific spectral distributions. Therefore, this paper applies a two-dimensional discrete Fourier transform to feature maps or image patches. Let the input signal be u(p,q); its two-dimensional frequency-domain representation is

(6) $$F\left(m,n\right)=\sum_{p=0}^{M-1}\sum_{q=0}^{N-1}u\left(p,q\right)\exp\left(-j2\pi \left(\frac{mp}{M}+\frac{nq}{N}\right)\right)$$

where (p,q) denotes spatial coordinates, (m,n) denotes frequency indices, and *M* and *N* denote the size of the spectrum. The corresponding magnitude spectrum is

(7) $$A\left(m,n\right)=\left|F(m,n)\right|$$

To reduce scale differences, a logarithmic transformation is further applied:

(8) $$\tilde{A}\left(m,n\right)=\log\left(A(m,n)+\delta \right)$$

where δ is a very small constant used to avoid logarithmic overflow. Let ${\tilde{A}}_{gen}$ and ${\tilde{A}}_{r}$ denote the log-magnitude spectra of the generated sample and the reference sample, respectively. The frequency-domain consistency loss is then defined as

(9) $$L_{freq}=∥{\tilde{A}}_{gen}-{\tilde{A}}_{r}∥$$

This term aligns the energy distributions of the generated sample and the reference defect sample in the frequency domain, rather than only comparing pixel intensities. It can, therefore, constrain the underlying frequency structure of textures more directly. Because the generated image should also remain globally consistent with the target background, a content constraint term is added:

(10) $$L_{content}={∥T\left(d(Z_{t})\right)-T\left(X^{n}\right)∥}_{1}$$

where T(·) denotes a content feature extractor, which is usually chosen as a shallow visual feature map. The frequency-guidance energy function in the multi-domain consistency module can, therefore, be written as

(11) $$\psi \left(Z_{t}\right)=L_{sink}+\lambda_{f}^{\left(1\right)}L_{freq}+\lambda_{f}^{\left(2\right)}L_{content}$$

where $\lambda_{f}^{\left(1\right)}$ and $\lambda_{f}^{\left(2\right)}$ are the weights of the frequency-domain term and the content term, respectively.

With this design, frequency-domain consistency guidance no longer relies only on the noise-prediction ability implicitly learned by the diffusion model. Instead, it actively guides the generated results toward directions that better match the spectral distribution of real defects. This improves texture detail in defect regions and reduces local texture smoothing and edge distortion, thereby providing a stronger realism basis for later background blending and structural control.

#### 3.2.2. Cross-Domain Context-Adaptive Fusion Mechanism

**Figure 3:**
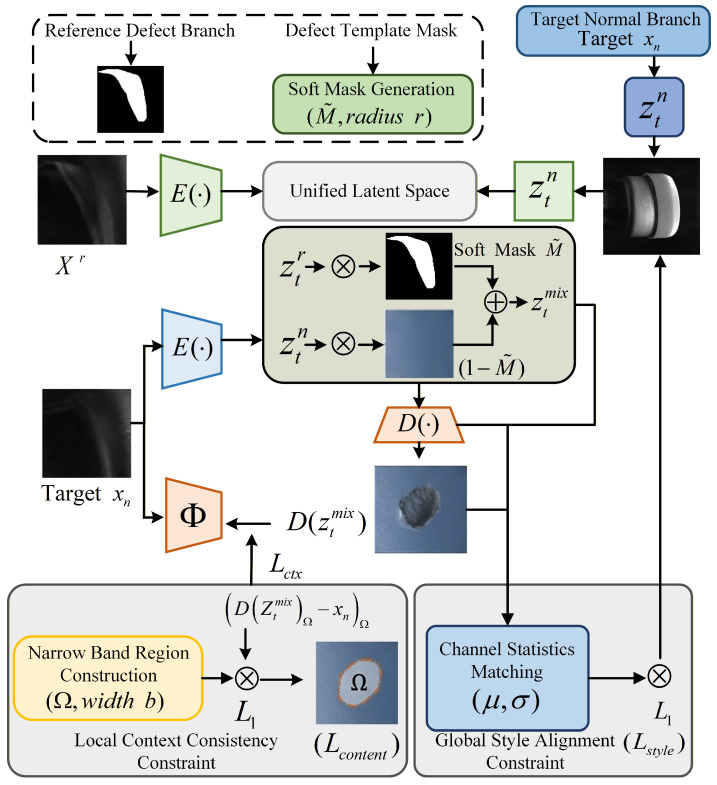
Mechanism of cross-domain context-adaptive fusion.

Even when the frequency structure of defect textures is improved, the generated result may still appear unnatural because of background-domain differences. In industrial scenarios, reference defect samples and target normal samples often come from different devices, lighting conditions, or production batches. These differences appear not only in brightness and color, but also in local background texture, surface reflections, and noise statistics. If defect generation simply places the reference anomaly into the target image without considering the context around the anomalous region, the final result may show obvious collage-like artifacts at the boundary. To address this issue, this paper further proposes a cross-domain context-adaptive fusion mechanism. As shown in Figure 3, the defect generation process is modeled as a conditional fusion process constrained by target background statistics.

Let the defect-region template be *M*. A continuous soft mask $\tilde{M}$ is obtained by dilation, erosion, and Gaussian smoothing with radius r. At the t-th reverse denoising step, let $Z_{t}^{r}$ denote the latent variable predicted by the reference-defect branch and $Z_{t}^{n}$ denote the latent variable of the target-normal branch. Their context-adaptive fusion is then written as

(12) $$Z_{t}^{mix}=\tilde{M}⊙Z_{t}^{r}+\left(1-\tilde{M}\right)⊙Z_{t}^{n}$$

where $Z_{t}^{mix}$ denotes the fused latent variable. Equation (12) does not combine arbitrary latent variables directly. Instead, it is defined in a unified latent representation space. Because the two branches are produced by the same encoder and diffusion backbone, their channel semantics and spatial arrangement are consistent. Therefore, this linear combination can be regarded as conditional interpolation on a shared latent semantic basis rather than a mechanical combination of incomparable representations.

To obtain more natural local texture transitions between defect boundaries and the target background, this paper defines a narrow-band region Ω around the boundary of the soft mask with width *b*, pixels and imposes a local context consistency constraint in feature space. Let Φ(·) denote a pretrained visual encoder. The local context loss is defined as

(13) $$L_{content}={∥\Phi {\left(d\left(Z_{t}^{mix}\right)\right)}_{\Omega }-\Phi {\left(X^{n}\right)}_{\Omega }∥}_{\;\;\;\;\;1}$$

This loss acts only on the defect boundary and its neighborhood rather than on the whole defect interior. Therefore, it does not over-homogenize the anomalous texture, but instead promotes natural blending in the boundary band.

To reduce global style shift, this paper further matches the channel statistics of the fused latent variable to those of the target-background latent variable. Let μ(·) and σ(·) denote the channel mean and standard deviation of a feature map along the spatial dimension, respectively. The style alignment loss is defined as

(14) $$L_{style}={∥\mu \left(Z_{t}^{mix}\right)-\mu \left(Z_{t}^{n}\right)∥}_{1}+{∥\sigma \left(Z_{t}^{mix}\right)-\sigma \left(Z_{t}^{n}\right)∥}_{1}$$

By combining frequency-domain consistency guidance and cross-domain contextual fusion, the multi-domain consistency constraint module is written as

(15) $$L_{cons}=\lambda_{c}^{\left(1\right)}L_{sink}+\lambda_{c}^{\left(2\right)}L_{freq}+\lambda_{c}^{\left(3\right)}L_{content}+\lambda_{c}^{\left(4\right)}L_{style}$$

where $\lambda_{c}^{\left(1\right)},\;\lambda_{c}^{\left(2\right)},\;\lambda_{c}^{\left(3\right)}$ and $\;\lambda_{c}^{\left(4\right)}$ are the weights of the corresponding terms. At this stage, realism constraints are no longer limited to spatial-domain feature similarity. They jointly cover frequency-domain texture structure, local boundary transition, and global imaging-style adaptation.

Using only frequency-domain constraints can improve texture energy distribution, but it cannot fully resolve collage-like artifacts caused by background-domain differences. Using only contextual fusion can improve local style consistency, but it cannot explicitly correct the spectral structure of defect textures. Therefore, this paper models the two together to balance texture realism and background adaptability.

### 3.3. Geometric Semantic Constraints

In industrial defect image generation, it is not enough to produce images that only look like defects. It is also necessary to control how defects appear and where they appear. The first concerns geometric structural constraints, and the second concerns semantic spatial constraints. Without fine-grained modeling of these two aspects, generated defects may look realistic in local texture but still show contour distortion, unstable shape, or unreasonable placement. Based on this observation, this paper constructs geometric semantic constraints to improve generation controllability from both geometric priors and semantic region anchoring.

#### 3.3.1. Shape Constraint Mechanism Guided by Geometric Priors

In existing training-free generation methods, defect shape is usually controlled coarsely through reference templates and local masks. Although this can specify the general range of anomalies, it cannot constrain contour evolution continuously and, therefore, often produces boundary drift, local deformation, and contour breakage during diffusion sampling. For industrial defects, unstable shape directly weakens the structural rationality of generated samples and reduces their value as training data. To address this issue, this paper converts a discrete defect template into a continuous differentiable geometric prior and imposes shape constraints throughout sampling, so that the generated defect remains close to the reference anomaly in overall contour and boundary trend. The proposed shape constraint does not force the generated defect to copy the reference template exactly. Instead, it jointly constrains coarse shape trend through low-frequency contour consistency and boundary-gradient consistency, leaving room for local detail variation while preserving category-level topological characteristics.

**Figure 4:**
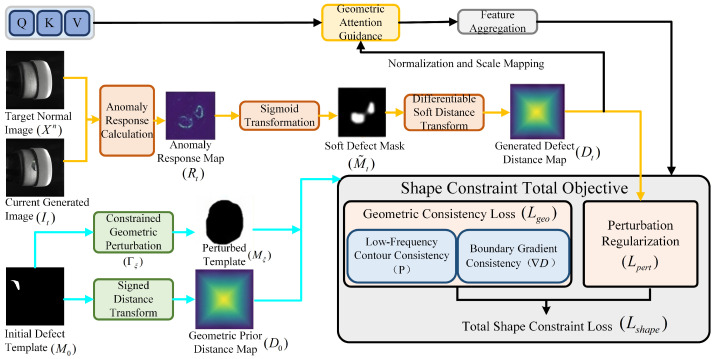
Mechanism of shape constraint guided by geometric priors.Different colored arrows are used to distinguish different processing paths in the proposed mechanism and do not indicate quantitative differences.

As shown in Figure 4, let the initial defect template $M_{0}$ be a binary mask, where $M_{0}\left(i,j\right)=1$ indicates the defect region and $M_{0}\left(i,j\right)=0$ indicates the background. To convert this discrete template into a continuous geometric prior, a signed distance transform is first applied to $M_{0}$ to obtain the distance map

(16) $$D_{0}\left(i,j\right)=\left\{\begin{array}{cc} dist\left(\left(i,j\right),\partial M_{0}\right), & \left(i,j\right)\in M_{0}\\ -dist\left(\left(i,j\right),\partial M_{0}\right) & \left(i,j\right)\notin M_{0} \end{array}\right.$$

where $\partial M_{0}$ denotes the template boundary and dist(·) denotes the Euclidean distance. This representation not only distinguishes the interior from the exterior but also encodes the distance of each position to the target contour. It is, therefore, more suitable than a binary mask for use as a continuous differentiable shape prior.

In the attention layer, the distance map is converted into a geometric bias term and injected into the attention scores. Let the query, key, and value matrices be denoted by Q, K and *V*, respectively. The attention computation under geometric guidance is then formulated as

(17) $${\mathrm{Attention}}_{geo}\left(Q,K,V\right)=\mathrm{softmax}\left(\frac{QK^{⊤}}{\sqrt{d}}+\beta G\right)V$$

where *d* is the dimensionality of the key vectors; *G* denotes the bias matrix obtained by normalizing and scale-mapping the prior distance map and β is the geometric-guidance strength coefficient. Unlike hard-mask feature cropping, Equation (17) constrains defect shape by modifying the attention distribution. This encourages the model to aggregate features along more plausible contour evolution paths instead of mechanically copying a fixed boundary.

To construct a differentiable soft defect-shape representation from the current generated result, let $I_{t}$ be the intermediate generated image at the current time step and $X^{n}$ be the target normal image. The anomaly response map is defined as

(18) $$R_{t}=\varsigma \left(\left|I_{t}-X^{n}\right|\right)$$

where ‖ denotes the pixel-wise absolute value and ς(·) denotes a response-mapping function composed of continuous operations such as smoothing and normalization. By further applying transformation to this response map, the soft defect mask at the current time step can be obtained as

(19) $${\tilde{M}}_{t}=\mathrm{sigmoid}\left(k\left(R_{t}-\delta \right)\right)$$

where *k* is the slope parameter and δ is the threshold. Because the above operations are continuous and differentiable, they can serve as intermediate variables in the geometric consistency constraint. Applying the same soft distance transform to ${\tilde{M}}_{t}$ yields the distance map of the generated defect $D_{t}$.

The geometric consistency loss is then defined by comparing the generated distance map with the prior distance map in terms of low-frequency contours and local gradients:

(20) $$L_{geo}={∥P\left(D_{t}\right)-P\left(D_{0}\right)∥}_{1}+\lambda_{g}{∥\nabla D_{t}-\nabla D_{0}∥}_{1}$$

where P(·) denotes a low-frequency projection operator used to extract coarse contour information from the distance map; ∇ denotes the spatial gradient operator and $\lambda_{g}$ is the weight of the gradient term. The first term keeps the generated defect consistent with the prior template at the coarse contour level, while the second term limits boundary deviation without rigidly fixing all details. This leaves room for local elastic variation.

To avoid rigid modes caused by a fixed template, this paper further introduces constrained geometric perturbation. Let $\Gamma_{\xi }\left(\cdot \right)$ denote a random geometric transformation with parameter ξ, including lightweight affine transformation and elastic deformation. The perturbed template is written as

(21) $$M_{\xi }=\Gamma_{\xi }\left(M_{0}\right),\;\xi ∼p\left(\xi \right)$$

To prevent excessively large transformations from destroying the topological semantics of the original defect category, a perturbation regularization term is introduced:

(22) $$L_{pert}={∥\xi ∥}_{2}^{2}$$

Accordingly, the overall objective of the shape-constraint module can be written as

(23) $$L_{shape}=L_{geo}+\lambda_{p}L_{pert}$$

where $\lambda_{p}$ is the weight of the regularization term. At this point, the geometric prior not only specifies what the defect should roughly look like but also allows reasonable instance-level variation.

By introducing a continuous distance-field prior and a geometric consistency constraint, the proposed method stabilizes the contour trend of defects without making the template overly rigid. As a result, generated anomalies better follow the geometric structure of reference defects in both overall shape and boundary evolution. This design reduces contour drift and local deformation and also provides a more stable basis for subsequent spatial semantic control.

#### 3.3.2. Semantic Region-Anchored Attention Regulation Mechanism

**Figure 5:**
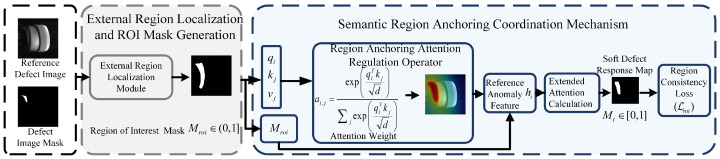
Mechanism of semantic region-anchored attention regulation.

Ensuring reasonable shape alone is still insufficient for practical industrial scenarios. Real defects usually have clear spatial attachment relationships, and different anomaly categories often appear only on specific components or within specific regions. If a generated defect has a plausible contour but appears in an implausible location, it still violates industrial logic. This problem is especially evident in transparent-container and complex-component scenarios, where the distinction between background regions and structural regions is clearer and cross-boundary anomalies are more conspicuous. To address this issue, this paper further introduces a semantic region-anchored attention regulation mechanism. This mechanism constrains where reference anomaly information can be injected through regional priors, so that defect generation is controlled not only by local appearance but also by component-level semantic support-domain constraints, as shown in Figure 5.

Let the region-of-interest mask obtained by the external region localization module be $M_{roi}\in {\left[0,1\right]}^{H\times W}$, where *H* and *W* denote the height and width of the current feature map, respectively. Before use, the mask is aligned with the current feature resolution, and $M_{roi}^{\left(i\right)}$ denotes the mask value at the i-th spatial position. A larger value indicates a higher $M_{roi}^{\left(i\right)}$ probability that the position belongs to the target semantic region in which defect generation is allowed. It should be noted that the ROI mask is an external spatial prior used only during inference. It is generated by the region localization module according to the anomalous location in the reference defective image and determines the region where anomaly injection is allowed. In our implementation, the ROI prior is annotation-derived rather than produced by an additionally trained detector. For the MVTec AD dataset, it is obtained from the official pixel-level anomaly mask of the selected reference defect sample; for the self-constructed vial dataset, it is obtained from the manually annotated defect mask. The connected anomalous region in the reference mask is extracted, slightly expanded, and resized to the current feature resolution to form the ROI mask. Neither the diffusion backbone nor the guidance branch is updated on the target task, and the ROI mask does not participate in parameter optimization. Therefore, introducing this prior does not change the training-free property of the proposed method [28].

On this basis, the semantic regional prior is introduced into the cross-attention process. Let the query vector at query position be $q_{i}\in R^{d}$, and let the key and value vectors at position j in the reference feature be $k_{j}\in R^{d}$ and $v_{j}\in R^{d}$, respectively. Here, *d* denotes the dimensionality of the query, key, and value vectors. The basic attention weight from position *i* to each reference position is defined as

(24) $$a_{i,j}=\frac{\exp\left(\frac{q_{i}^{⊤}k_{j}}{\sqrt{d}}\right)}{\sum_{j}\exp\left(\frac{q_{i}^{⊤}k_{j}}{\sqrt{d}}\right)}$$

where $q_{i}^{T}k_{j}$ denotes the correlation between query position and reference position; $\sqrt{d}$ is a scaling factor and $\sum_{j}$ denotes normalization over all reference positions. Thus, $a_{i,j}$ represents the relative weight with which query position *i* receives information from each reference position.

To make the semantic constraint regulate whether the current position receives anomalous information, rather than changing the relative ranking of different reference tokens under the same query position, this paper further introduces regional gating in the attention aggregation stage. The semantically anchored anomalous feature representation is then written as

(25) $$h_{i}={\left(R_{i}+\varepsilon \right)}^{\alpha }\sum_{j}a_{i,j}v_{j}$$

where $h_{i}$ denotes the cross-attention output feature at position after semantic regional constraint, and $\sum a_{i,j}v_{j}$ denotes the standard cross-attention aggregation result; ε>0 is a very small constant used for numerical stability and α>0 is the regional anchoring strength parameter. When $M_{roi}^{\left(i\right)}$ is large, the current position can receive reference anomalous information more fully. When it is small, anomaly injection is globally suppressed. In this way, $h_{i}$ is used as one of the inputs to the subsequent diffusion denoising process and further affects the anomaly response distribution at the current time step.

To further prevent generated anomalies from crossing boundaries at the output level, let ${\tilde{M}}_{t}\in R^{H\times W}$ be the soft defect response map predicted at the current time step. The region consistency loss is defined as

(26) $$L_{roi}=∥\left(1-M_{roi}\right)⊙{\tilde{M}}_{t}∥$$

where $1-M_{roi}$ denotes the complementary region of the ROI mask, that is, the non-target semantic region; ⊙ denotes element-wise multiplication and ‖ ‖ denotes the $L_{1}$ norm operation. This loss penalizes anomalous responses only in non-target regions and suppresses defect overflow at the output-distribution level. Therefore, Equation (25) constrains where anomalous information can be injected during feature transmission, whereas Equation (26) constrains the spatial range of anomaly distribution at the output stage. Together they form a two-level regional control mechanism from intermediate features to final outputs.

By combining the region-anchored guidance term and the region consistency constraint, the semantic constraint objective can be written as

(27) $$L_{sem}=\lambda_{a}L_{anchor}+\lambda_{r}L_{roi}$$

where $L_{sem}$ denotes the total semantic constraint loss, $L_{anchor}$ denotes the region-anchored attention guidance term used to ensure effective injection of anomalous features inside the target region, and $L_{roi}$ denotes the out-of-boundary suppression term defined in Equation (26). $\lambda_{a}$ and $\lambda_{r}$ are the corresponding weights. This expression indicates that the semantic constraint not only requires effective anomaly generation within the target region, but also requires suppression in non-target regions.

By further combining the shape constraint and the semantic regional constraint, the overall objective of the geometric semantic constraint mechanism is obtained as

(28) $$L_{struct}=\lambda_{sg}L_{shape}+\lambda_{ss}L_{sem}$$

where $L_{shape}$ denotes the shape constraint term used to restrict the geometric contour and local morphology of defects, while $\lambda_{sg}$ and $\lambda_{ss}$ control the trade-off between geometric structural constraints and spatial semantic constraints, respectively. Equation (28) shows that the proposed control mechanism does not repeatedly model the same problem. Instead, it imposes complementary constraints from two aspects: how defects should grow and where defects should grow.

Through the joint constraints of region-anchored attention regulation and region consistency loss, the proposed method can more stably confine anomalies within the target support domain and suppress anomalous responses in non-target regions. This reduces positional drift and out-of-boundary phenomena and makes the spatial distribution of generated defects more consistent with the structural logic of industrial objects. As a result, the controllability and task adaptability of the generated samples are further improved.

## 4. Experiments

### 4.1. Experimental Settings

#### 4.1.1. Datasets and Annotation Settings

**Figure 6:**
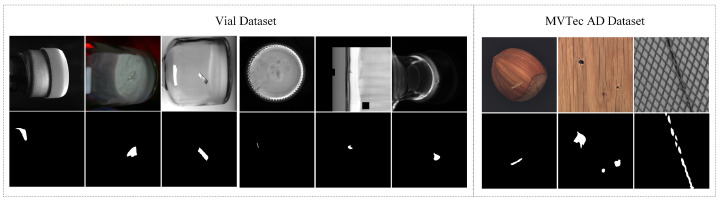
Examples from the self-constructed vial defect dataset and the MVTec AD dataset. The left panel presents representative samples from the vial defect dataset together with their corresponding annotations, covering typical defect locations including cap appearance, cake surface, bottle appearance, bottle bottom, bottle expansion, and cap edge; the right panel shows representative samples and annotations from the MVTec AD dataset.

Experiments are conducted on the MVTec AD dataset and a self-constructed vial defect dataset, as shown in Figure 6. MVTec AD is one of the most widely used public benchmarks in industrial anomaly detection [28]. It contains 15 categories of industrial images that cover both texture and object characteristics and can effectively reflect the applicability of a method in standard industrial scenarios. In the quantitative comparison, all 15 categories are used for evaluation. In the analysis, representative categories such as grid, hazelnut, and wood are further examined to verify the applicability of the proposed method in standard industrial scenarios [29].

**Figure 7:**
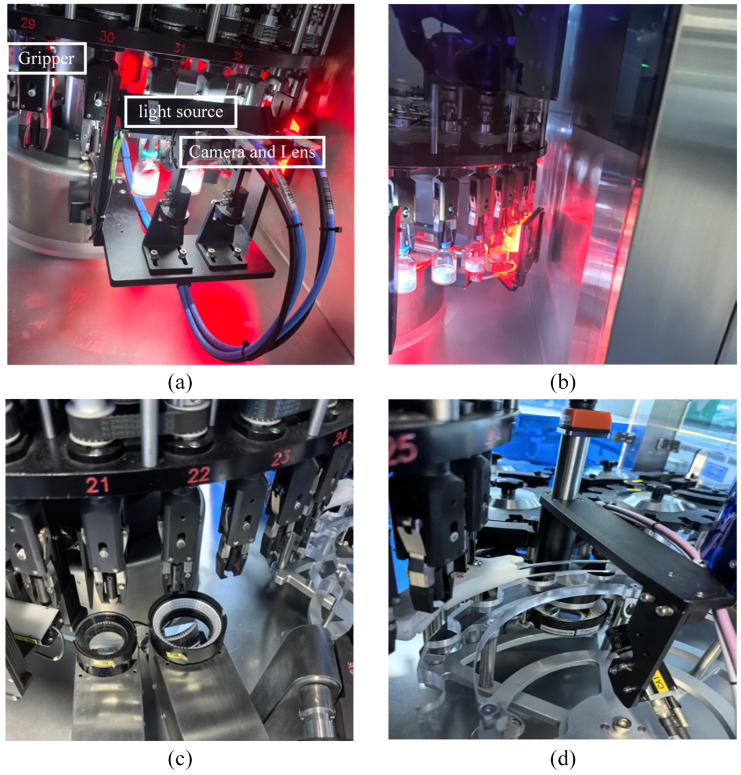
Multi-view photographs of the vial visual inspection system. The system consists of a conveyor line, rotary clamping mechanism, illumination unit, industrial camera, lens, and host platform, supporting online image acquisition for subsequent defect annotation, anomalous sample generation, and downstream detection. (**a**) Annotated overview of the imaging workstation, including the gripper, light source, camera, and lens; (**b**–**d**) additional views of the actual acquisition environment from different angles, illustrating the mechanical positioning structure, illumination condition, and local imaging workspace.

To verify the background blending and structural control capability of the proposed method under complex imaging conditions, a self-constructed vial defect dataset is further established in addition to the MVTec AD dataset. To meet the practical requirements for high-precision and high-efficiency vial appearance inspection in pharmaceutical production, an online image acquisition system based on machine vision is constructed to collect vial sample images under real quality inspection conditions. The multi-view photographs of the vial visual inspection system are shown in Figure 7, and the parameters of the related acquisition equipment are listed in Table 1.

**Figure 8:**
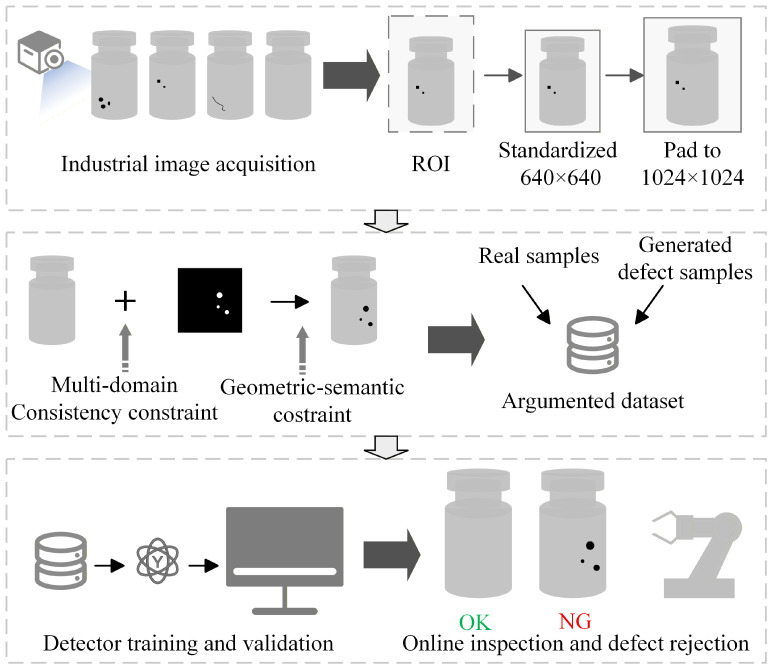
Workflow of the vial visual inspection system. OK indicates qualified vials, whereas NG indicates defective vials.

The system simulates the visual inspection workflow of an actual pharmaceutical production line, as shown in Figure 8. Vials to be inspected first enter the clamping station along the conveyor line, where the rotary clamping mechanism ensures continuous and stable automated handling and accurately transfers the vials into the illumination area. Under the combined illumination of a high-intensity ring light and auxiliary light sources, the industrial camera is synchronized with the rotation of the clamping mechanism to capture high-resolution images of key structural regions of the vials. The acquired image data are then uploaded to the host platform for subsequent defect annotation, anomalous sample generation, and downstream detection model training and evaluation.

Considering that vials are transparent glass containers with reflection, specular highlights, and edge refraction, high-quality labels were constructed through manual annotation. During annotation, professional image annotation tools were used to provide pixel-level masks for typical defects such as fine cracks, glass burrs, scratches, and stains on the vial surface. Meanwhile, tight object-detection bounding boxes enclosing the defect regions were generated based on the corresponding masks. This refined dual-annotation scheme provides accurate spatial references for anomaly-region guidance and structural constraints and also supports the training and evaluation of downstream detectors. The self-constructed vial dataset contains six defect categories, with 500 anomalous images for each category and 3000 anomalous images in total. In addition, normal vial images were collected from the corresponding key inspection regions. Specifically, 1000 normal images were collected for each inspection region, resulting in 6000 normal images in total. Therefore, the normal/anomalous sample ratio of the self-constructed vial dataset is 2:1. The six defect categories correspond to six key-location anomalies: Bottle Expansion (BE), Bottle Bottom (BB), Cap Edge (CE), Cake Surface (CS), Cap Appearance (CA), and Bottle Appearance (BA). The normal images are used as target background images during defect generation, while the anomalous images and their masks provide reference defect information for anomaly synthesis. The raw images acquired by the industrial camera are initially captured at 1920 × 1200 pixels. For subsequent experiments, the images are cropped around defect-related regions and resized to 640 × 640, which is used as the input resolution of the downstream detector. According to Table 2, the dataset is divided into training, validation, and test sets to ensure consistent training and evaluation. To avoid data leakage, the reference defect images and their corresponding masks used during defect generation are selected only from the training split, while the final test images are kept strictly independent. No reference defect image or reference mask used for generation is reused as a final test sample in downstream detection evaluation.

To further illustrate the complexity of the self-built vial defect dataset, this paper conducts a statistical analysis of the morphological and scale characteristics of six typical defect categories, as shown in Table 3. The statistical indicators include the average aspect ratio, average area, and the dispersion degrees of the aspect ratio and area of defect regions. Among them, the average aspect ratio is used to reflect the spatial extension direction of defect regions, the average area is used to characterize the defect scale, and the dispersion degree is used to measure the stability of morphological and scale variations within the same category.

From the overall results, different defect categories exhibit obvious differences in scale range, morphological extension direction, and distribution stability. This indicates that vial defects are not conventional anomalies with a single scale or a single texture pattern, but instead include multiple cases such as tiny defects, structural defects, and large-area regional anomalies. Such complexity increases the difficulty of both defect generation and downstream detection tasks and also imposes higher requirements on the texture realism, morphological rationality, and spatial controllability of generated samples.

Specifically, Cap Appearance defects have a relatively large average aspect ratio, showing obvious vertical elongation characteristics. Bottle Appearance defects have a relatively small average aspect ratio and are closer to horizontal or block-like distributions. Cap Edge and Cake Surface defects exhibit moderate morphological elongation. In terms of area statistics, Cap Edge defects have a significantly larger average area than the other categories, indicating that this type of defect usually covers a wider region. By contrast, Bottle Bottom and Bottle Expansion defects have smaller average areas and are closer to fine-grained defects. Their generation and detection, therefore, rely more heavily on local texture details, edge information, and high-resolution feature representation.

The above statistical results show that the self-built vial dataset contains multiple defect categories with significant scale differences, complex structural morphologies, and imbalanced inter-category distributions. This characteristic further demonstrates that relying solely on local anomalous appearance transfer is insufficient to cover the complex distribution of real industrial defects. Therefore, this paper introduces multi-domain consistency constraints into the defect generation process to enhance the realism of defect textures, boundary transitions, and background integration. Meanwhile, geometric-semantic constraints are introduced to improve the rationality of defect shape evolution and spatial placement. Table 3 not only provides supplementary evidence for the morphological and scale characteristics of the self-built dataset, but also offers a data basis for the subsequent analysis of generation quality and detection performance differences among different defect categories.

The original MVTec AD dataset mainly provides image-level and pixel-level anomaly annotations, but it does not directly provide object-detection bounding boxes. To construct the downstream detection task, this paper converts the official pixel-level anomaly masks into bounding-box annotations. Specifically, connected-component analysis is first applied to each anomaly mask. Then the minimum enclosing rectangle of each connected anomalous region is extracted as the object-detection label. Small noisy regions with areas below a threshold are filtered out to reduce interference from pseudo-targets during detector training. For generated samples, the corresponding bounding-box labels are synchronously generated from anomaly masks in the same way, which ensures a consistent task definition for downstream detection experiments across different augmentation methods.

#### 4.1.2. Experimental Environment

The experimental platform is configured with an Intel Core i7-13700F CPU, 32 GB memory, and an NVIDIA GeForce RTX 4090 GPU. The software environment includes Windows 10, Python 3.9, PyTorch 2.0, and CUDA 11.8. To ensure fair comparison, YOLOv11 is uniformly selected as the downstream detector. The data split, number of training epochs, learning rate, optimizer, and input resolution are kept the same across all methods, and the only difference is the source of the training samples. To balance the preservation of real data distribution and the effectiveness of defect-sample augmentation, the ratio of original samples to synthetic samples is uniformly set to 1:1 for all methods. Detailed settings are listed in Table 2.

To further reduce bias caused by differences in sample scale, all augmentation methods generate the same number of synthetic samples, and only the generation strategy differs. Specifically, in the vial scenario, the algorithm generates 1000 pairs of anomalous samples with high-precision mask annotations for each defect category for generation-quality evaluation and downstream detection experiments. On the MVTec AD dataset, each category also uses a fixed number of augmented samples. The main purpose is to compare generation quality across methods in standard industrial scenarios.

In the data preprocessing stage, images used in the generation branch are adjusted to a fixed resolution of 1024 × 1024 using a unified padding strategy to meet the input requirements of the pretrained diffusion model. It should be noted that 640 × 640 is used as the standardized resolution for the processed dataset and the input size of the downstream detector, whereas 1024 × 1024 is used only in the generation branch to satisfy the input-resolution requirement of the diffusion model. Here, 640 × 640 corresponds to the original data resolution and the input size of the downstream detector, whereas 1024 × 1024 is used only in the generation branch to satisfy the input-resolution requirements of the diffusion model. To preserve fine details in vial images, the algorithm first performs local cropping and scale enlargement on potential defect regions with the help of annotation masks. This local-focusing strategy retains as many fine defect details as possible, such as cracks, under limited computational resources. Through this standardized preprocessing pipeline, the generated defect images maintain high semantic consistency with the original annotations and better match the practical requirements of high-precision industrial inspection.

#### 4.1.3. Implementation Details

The proposed method is built upon the TF-IDG framework; therefore, the overall diffusion backbone and inference pipeline follow the settings of the original framework. Specifically, pretrained Stable Diffusion and its corresponding ControlNet are adopted for image editing, without any parameter updates. The ROI is obtained before image generation and is used only as a spatial constraint for defect generation during the inference stage. Feature representations are extracted by the pretrained feature encoder in the TF-IDG framework, while the proposed method is used only to guide the diffusion sampling process. The sampling strategy follows the same DDIM Scheduler configuration as the original framework. During the entire inference stage, the inputs include a normal image, a reference defective image, the corresponding defect mask, and the ROI prior, without the need to train any additional model.

For hyperparameter settings, the newly introduced constraint terms use fixed weights, and the detailed values are listed in Table 4. Here, MDC denotes the multi-domain consistency constraint mechanism, and SSC denotes the geometric semantic constraint mechanism.

To prevent scale differences among different constraint terms from affecting the generation process, this paper determines the weight parameters by combining empirical tuning through repeated experiments with grid search. Specifically, the parameter search ranges are first determined by referring to the magnitude settings of the energy guidance terms in the original TF-IDG framework. Then, grid search is performed on the validation set for the weights of the multi-domain consistency constraints and geometric-semantic constraints, respectively, with a step size of 0.05. Local IS, IC LPIPS, and downstream detection mAP are used as comprehensive evaluation metrics. All experiments maintain the same parameter configuration, and no category-specific parameter tuning is conducted, so as to ensure fairness. When further parameter adjustment no longer brings obvious performance improvement, the final configuration is adopted as the experimental setting of this paper. Therefore, the parameters listed in Table 4 are fixed values obtained after validation-set tuning.

Given the need for fine-grained structure preservation and local texture control in industrial defect generation, the denoising process adopts a DDIM scheduler with 50 sampling steps. The first 30 steps perform feature alignment and gradient guidance, and the last 20 steps perform texture enhancement and detail refinement. In addition, the ROI priors are obtained by a region localization module based on the positions of reference defect images, and its parameters are not used to update the defect-generation backbone. In the vial scenario, the ROI can be directly obtained from component-region annotations or an existing localization module [28,30].

### 4.2. Evaluation Metrics

This paper evaluates the proposed method from three aspects: generation quality, sample diversity, and downstream task gain. The first two metrics reflect the realism and diversity of the generated samples, while the last directly measures the practical value of the augmented samples for defect detection.

Because industrial defect generation focuses more on the quality of local anomalous regions than on the global naturalness of the whole image, this paper adopts the Local Inception Score (Local IS) to measure local realism. Its mathematical expression is [31]

(29) $$\mathrm{Local}\;\mathrm{IS}=\exp\left(E_{x∼P_{g}}\left[D_{KL}\left(p(y|x)\|p(y)\right)\right]\right)$$

where E denotes expectation; the subscript $x∼P_{g}$ indicates the corresponding metric values for all defect samples under the generated-sample distribution; *x* denotes the local defect patch cropped from the masked region of a generated image; p(y∣x) denotes the conditional probability distribution output by a pretrained classification network for the local defect patch; p(y) denotes the marginal distribution of the full set of generated local samples in the category space and $D_{KL}$ denotes the KL divergence. A larger Local IS indicates that the generated defects are more realistic and have stronger category discriminability.

To quantitatively evaluate sample diversity under complex backgrounds and to prevent the model from producing repeated or overly similar defect patterns, this paper further introduces the intra-class diversity metric IC LPIPS based on perceptual similarity [28,32]. This metric measures perceptual differences among generated samples within the same defect category. Its calculation is

(30) $$\mathrm{IC}\;\mathrm{LPIPS}\left(x_{i},x_{j}\right)=\sum_{l}\frac{1}{H_{l}W_{l}}\sum_{h,w}{∥w_{l}⊙\left({\hat{f}}_{l}{\left(x_{i}\right)}_{hw}-{\hat{f}}_{l}{\left(x_{j}\right)}_{hw}\right)∥}_{2}^{2}$$

where *l* denotes the feature layer of a deep convolutional network; $H_{l}$ and $W_{l}$ denote the height and width of the corresponding feature map, respectively, *h* and *w* denote the spatial position indices along the height and width of the *l*-th feature map; ${\hat{f}}_{l}\left(\cdot \right)$ denotes normalized feature activation and $w_{l}$ denotes the weighting vector for feature differences at that layer. By averaging this distance within the same defect category, a larger IC LPIPS indicates stronger intra-class variation and thus better diversity.

To directly verify the practical support of generated samples for downstream detectors, this paper adopts Precision (P), Recall (R), and AP from object detection as evaluation metrics. These metrics are used to measure the effect of different augmentation methods on detection performance. Precision represents the proportion of true positives among the predicted positives. Recall represents the proportion of real targets that are correctly detected. mAP@50 denotes the mean average precision across categories under the condition that IoU is no less than 0.5. Their definitions are

(31) $$P=\frac{TP}{TP+FP}$$

(32) $$R=\frac{TP}{TP+FN}$$

(33) $$AP=\int_{0}^{1}P\left(R\right)\;dR$$

(34) $$mAP=\frac{1}{N}\sum_{i=1}^{N}AP_{i}$$

where *N* denotes the number of defect categories, and $AP_{i}$ denotes the average precision of the *i*-th defect category under the condition IoU >= 0.5.

### 4.3. Comparative Experiments

The proposed method is compared with Cut-Paste, DFMGAN, AnoDiff, AnoAny, and the original TF-IDG on both the MVTec AD dataset and the self-constructed vial defect dataset.

#### 4.3.1. Generation Quality and Diversity

Table 5 shows that the proposed method outperforms the comparison methods on most categories of the MVTec AD dataset. Its average IS and IC values reach 2.23 and 0.39, respectively, which are the best results among all methods. Compared with TF-IDG, these values improve from 2.14 and 0.36 to 2.23 and 0.39. This indicates that the generated samples are improved in both anomalous semantic expression and intra-class diversity and can provide clearer defects with more reasonable variation.

Looking at individual categories, the proposed method achieves the best IS values on bottle, cable, capsule, grid, hazelnut, metal nut, pill, tile, toothbrush, transistor, and zipper, with particularly clear gains on categories such as tile, grid, and pill. For example, on tile, IS and IC improve from 2.73 and 0.58 under TF-IDG to 2.89 and 0.63. On grid, they improve from 2.37 and 0.46 to 2.52 and 0.50. These results show that the proposed method is more effective for categories with complex textures and strong structural regularity.

The proposed method also shows advantages in diversity. The IC gains are especially clear on categories such as cable, grid, tile, transistor, and zipper. This indicates that the method can preserve instance-level variation while maintaining semantic consistency and is, therefore, closer to the distribution of real industrial defects. However, the proposed method is not optimal for every category. For example, on carpet, leather, screw, and wood, TF-IDG is slightly better on some metrics.

This suggests that the proposed improvements mainly target complex textured backgrounds, fine-grained boundary recovery, and insufficient spatial constraints. For categories with relatively stable textures and limited anomaly-shape variation, the gains may, therefore, be smaller.

Table 6 shows that the proposed method also achieves strong average performance on the vial dataset, with average IS and IC values of 2.34 and 0.32, respectively. Both values are higher than those of TF-IDG, which are 2.26 and 0.30. This indicates that the improved method has advantages in both generation quality and sample diversity.

At the category level, the proposed method achieves the best results on four defect categories: BE, CE, CA, and BA. The clearest improvement appears in CE, where IS increases from 2.39 to 2.56 and IC increases from 0.33 to 0.38. In BA, IS and IC increase from 2.27 and 0.29 to 2.45 and 0.34, respectively. These results show that the proposed method is more effective for defects with complex edge details and obvious local texture variation and can generate clearer and more realistic samples.

However, the proposed method is not the best in every category. For example, on BB and CS, TF-IDG achieves slightly higher values. This indicates that the original method already performs well on some categories with relatively stable structures and concentrated defect patterns, whereas the advantage of the proposed method is more evident in overall performance and on more complex categories. This trend is also consistent with practical observations in real industrial scenarios.

#### 4.3.2. Downstream Detection Performance

Table 7 and Table 8 show that detector performance on the MVTec AD dataset improves significantly after defect samples are augmented with generated images, and the proposed method achieves the best overall results.

For YOLOv11, the proposed method achieves average P, R, and AP values of 91.8%, 87.6%, and 89.6%, respectively. This is clearly better than the AP of 83.8% obtained without a generative model and also higher than the 88.5% AP of TF-IDG. The gains are more evident on categories such as cable, grid, screw, transistor, wood, and zipper. These categories usually involve elongated defects, complex textures, or strong background interference, and therefore, place higher demands on boundary quality and positional rationality. The proposed method provides more stable P, R, and AP values on these categories, which indicates that its generated samples are closer to the real defect distribution and are therefore more useful for detector training. For relatively easy categories such as bottle, hazelnut, and metalnut, the differences among methods are smaller, but the proposed method still maintains the best or near-best results.

Table 9 and Table 10 show that the proposed method yields even clearer improvements in downstream detection on the vial dataset. For YOLOv11, the average AP reaches 98.8%, which is higher than the 98.0% achieved by TF-IDG. This indicates that the generated anomalous samples are closer to the real defect distribution and can improve detector performance more effectively.

At the category level, CE shows the most significant improvement. For YOLOv11, its Recall increases from 68.0% without using a generative model to 89.8%, while AP increases from 85.6% to 94.9%. Compared with TF-IDG, Recall and AP still increase by 3.1 and 1.1 percentage points, respectively. Meanwhile, for categories such as CS and CA, whose performance is already close to saturation, the differences among methods are relatively small. Although the proposed method is not absolutely the best on every single metric, it still achieves the best or tied-best AP values. This indicates that the advantages of the proposed method are mainly reflected in overall performance improvement and in the improvement of more complex categories, making the experimental results more realistic and reasonable.

To evaluate the statistical stability of the downstream detection results, we repeated the key YOLOv11-based detection experiments three times under the same experimental protocol. The mean and standard deviation of mAP@50 are reported in Table 11. The results show that the proposed method consistently outperforms the baseline TF-IDG in repeated experiments on both datasets, and the performance fluctuation remains small. This indicates that the improvement brought by the proposed generation constraints is stable rather than caused by experimental randomness.

To further verify whether the samples generated by the proposed method can provide effective support for different downstream detectors, YOLOv26 was additionally adopted for supplementary validation after the YOLOv11-based downstream detection experiment. It should be noted that the YOLOv11 experiment was used as the main comparison among different augmentation methods, while the YOLOv26 [33] experiment was conducted to examine the detector-level applicability of the samples generated by the proposed method under another detection architecture.

As shown in Table 12 and Table 13, when YOLOv26 was used as another downstream detector, the proposed augmented dataset achieved an average precision of 93.5%, recall of 89.5%, and mAP@50 of 94.5% on the MVTec AD dataset. On the self-constructed vial defect dataset, YOLOv26 achieved an average precision of 98.1%, recall of 96.3%, and mAP@50 of 98.7%. These results show that the generated samples can still provide effective training support when a different detector is adopted. In particular, the supplementary YOLOv26 results further demonstrate that the proposed defect generation method has good detector-level applicability and practical value, rather than being effective only under a single downstream detection framework.

Overall, the results on the vial dataset further validate the effectiveness of the proposed method. It can provide higher-quality training samples for downstream defect detection and improve the overall detection performance of the model.

### 4.4. Ablation Study

To further analyze the contribution of each module to generation quality and downstream detection performance, this paper conducts ablation experiments on the vial dataset. The results are shown in Table 14. Here, FDG denotes frequency-domain consistency guidance, CAF denotes cross-domain context-adaptive fusion, ESC denotes elastic shape constraint, and SRA denotes semantic region-anchored attention regulation. FDG+CAF corresponds to the complete multi-domain consistency constraint, while ESC+SRA corresponds to the complete geometric semantic constraint. The original TF-IDG is used as the baseline method. Based on this baseline, progressive ablation configurations are constructed around the four submodules to examine the contribution of FDG, CAF, ESC, and SRA when they are introduced individually or gradually combined. The evaluation metrics include Local IS, IC LPIPS, and the mAP of the downstream detector YOLOv11.

The ablation results show that all four submodules improve generated sample quality and downstream detection performance to different degrees.

FDG and CAF mainly contribute to generation realism. FDG focuses more on improving the frequency structure and local texture expression of defect regions, whereas CAF focuses more on enhancing the naturalness of the transition between anomaly boundaries and the target background. Compared with the baseline TF-IDG, the relevant metrics show an upward trend when these two submodules are introduced individually. When they are used together, Local IS and YOLOv11 mAP further increase to 2.31 and 98.5, respectively. This indicates that the two submodules inside the multi-domain consistency constraint have good complementarity and can jointly improve the realism and stability of generated results.

On this basis, the introduction of ESC further enhances the stability of defect contour evolution, enabling the generated results to maintain more reasonable structural morphology while preserving texture realism. After SRA is further added, the complete model achieves the best performance. This indicates that spatial semantic constraints can further limit the diffusion range of anomalous responses and make defect placement more consistent with the structural distribution of target components.

Overall, the four submodules are not simply stacked. Instead, they produce a progressive collaborative effect at the two levels of realism enhancement and spatial control, which allows the complete model to achieve the best performance in both generation quality and downstream detection.

### 4.5. Generation Efficiency Analysis

This paper compares the inference efficiency of the original TF-IDG and the proposed method on the self-built vial dataset. The experiments are conducted under the same input resolution and the same number of sampling steps, where the generation resolution is set to (1024 × 1024), and the number of DDIM sampling steps is set to 50. The results are shown in Table 15.

Compared with the original TF-IDG, the proposed method reduces the peak GPU memory usage from 10.7 GB to 8.9 GB, the average generation time per image from 16.8 s to 15.2 s, and the intermediate attention cache from 2.41 GB to 1.63 GB. These results indicate that, under the same high-resolution generation setting, the proposed method does not introduce a significant additional inference burden after incorporating multi-domain consistency constraints and geometric-semantic constraints. Instead, it shows certain advantages in terms of GPU memory usage and average generation time. Since the proposed method does not require additional training of the diffusion backbone network, its computational cost is mainly concentrated in the inference stage. Therefore, it is more suitable for offline defect sample augmentation and training data construction in industrial vision-based sensing and inspection systems.

### 4.6. Visualization Analysis

To more intuitively show the differences in defect generation quality among different methods, this paper further provides a visualization analysis from four aspects: texture realism, naturalness of boundary blending, geometric contour preservation, and rationality of spatial placement.

**Figure 9:**
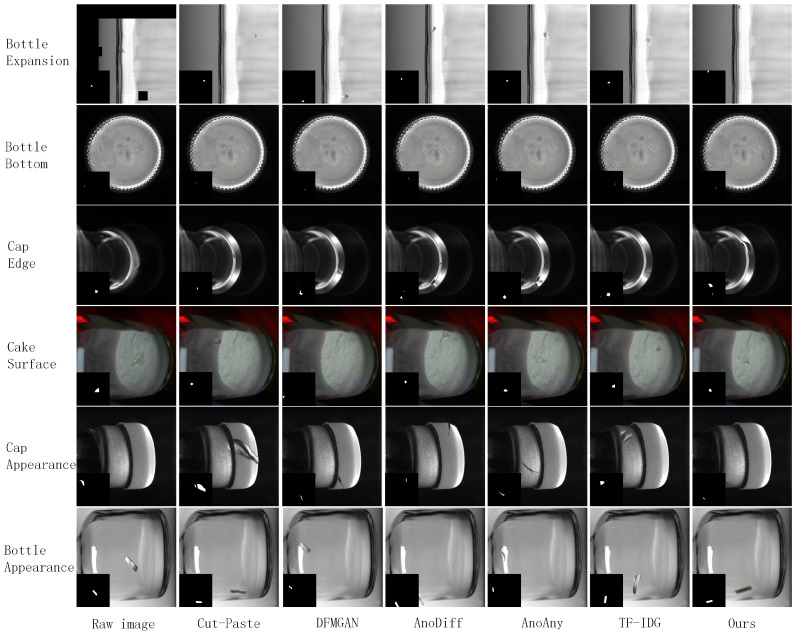
Visual comparison of different anomaly generation methods on the vial dataset.

First, in terms of texture realism, traditional copy-and-paste methods can generate anomalous samples quickly, but they often leave obvious splicing traces in texture details. This problem becomes more evident when the background contains reflections, high-frequency noise, or complex texture structures, where abrupt boundaries are likely to appear between anomalous regions and the original background. In contrast, diffusion-based generation methods can produce more natural anomalous textures at the overall visual level, but clear differences still exist among different methods. As shown in Figure 9, DFMGAN and AnoDiff can generate relatively clear anomalous regions in some samples, but their texture details still tend to be over-smoothed, and some local high-frequency structures are not sufficiently restored. AnoAny shows better semantic saliency in anomalous regions, but under complex background conditions, it may still produce unnatural boundary transitions. The proposed method can more completely recover fine-grained frequency components in defect textures in these samples, making the generated anomalies closer to real defects in visual quality. This advantage is especially evident for edge-type defects such as cracks, fine scratches, and glass burrs.

**Figure 10:**
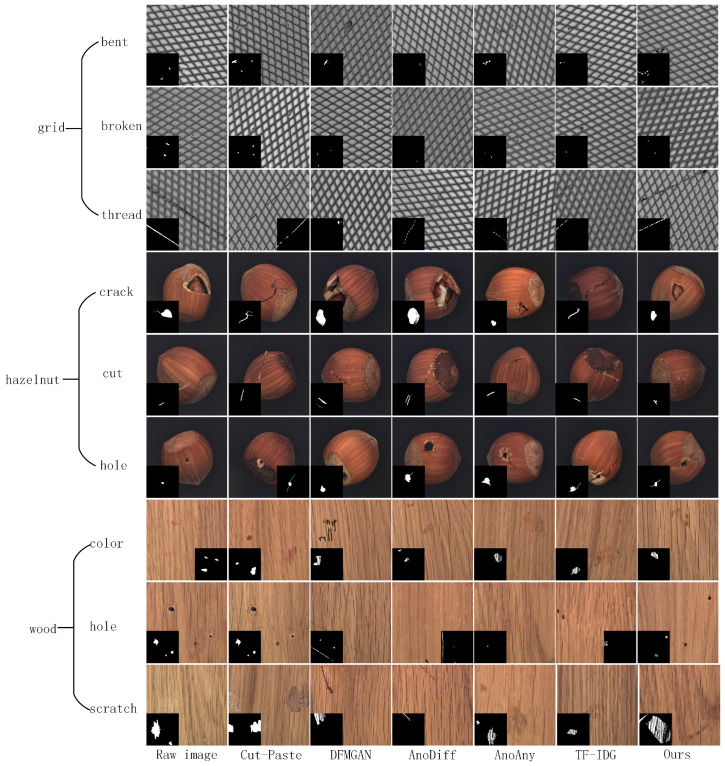
Visual comparison of different anomaly generation methods on the MVTec AD dataset.

Second, in terms of boundary blending, although the original TF-IDG can transfer reference defects to target images, it may still leave obvious transition discontinuities at defect boundaries due to the lack of consistency constraints on the contextual statistics of the target background. After introducing frequency-domain consistency guidance and cross-domain context-adaptive fusion, the boundary connection between the generated anomalies and the target background becomes smoother. As shown in Figure 10, the proposed method forms more continuous brightness and texture transitions around the outer edges of defects, thereby reducing the pasted-on appearance. This phenomenon indicates that the multi-domain consistency constraint not only improves the internal texture structure of defects but also enhances the overall coordination between anomalous regions and the background scene.

Third, in terms of geometric contour preservation, crack-like and scratch-like defects require high continuity in shape tendency. Without geometric-prior constraints, generated results are prone to main-contour bending, local shrinkage, or boundary fracture. As shown in Figure 9 and Figure 10, typical visual comparisons of geometric structures are presented. The original TF-IDG can generate anomalous regions in several samples, but its contours still deviate from the geometric tendency of the reference defects. After introducing elastic shape constraints, the proposed method better preserves the main contour direction and overall extension trend, making the anomalous regions closer to the natural growth pattern of real industrial defects.

Finally, in terms of spatial placement rationality, industrial defects usually have clear semantic attachment regions. If generated anomalies deviate from the target component or appear in implausible regions, they can hardly meet the logical requirements of industrial scenarios, even when their local textures appear realistic. The visualization results show that, under the constraint of ROI semantic anchoring, the proposed method can more stably confine anomalies within the target support domain, thereby reducing anomaly overflow and placement drift. This result is consistent with the performance improvement of position-sensitive defects such as CE in the quantitative experiments, indicating that the proposed method has good application value in controllability.

In summary, the qualitative analysis is consistent with the quantitative results. The proposed method shows better overall performance in defect texture realism, natural background blending, contour structure preservation, and spatial semantic rationality, which further validates the effectiveness of the multi-constraint collaborative framework.

## 5. Conclusions

### 5.1. Summary of the Work

To address sample scarcity, limited realism, and weak controllability in existing training-free industrial defect generation methods, this paper proposes a training-free industrial defect generation method based on multi-domain consistency and geometric semantic constraints. Built on the original TF-IDG framework, the proposed method improves both generation realism and generation controllability without additional training of the diffusion backbone. The definition of training-free in this paper follows that of the original TF-IDG paper, namely that the entire defect generation process is directly performed based on a pretrained diffusion model without updating the parameters of the diffusion model. The ROI required by the proposed method is obtained before image generation and is used only as a spatial constraint during the inference stage. It is not learned by the proposed method and does not participate in parameter optimization. Therefore, this prior does not change the training-free property inherited from TF-IDG.

The multi-domain consistency constraint focuses on texture frequency structure, boundary transition, and background blending, and improves the realism of generated defects. The geometric semantic constraint focuses on contour evolution and spatial placement and makes the generated results more consistent with real industrial patterns in both structure and region distribution. Experimental results on both the public MVTec AD dataset and the self-constructed vial dataset show that the proposed method performs well. Compared with the comparison methods, the generated samples show improvements in realism, diversity, and downstream detection utility. The ablation study further shows that the four submodules play different roles and work best when used together. These results indicate that the proposed method can achieve a good balance between generation quality and spatial control within a training-free framework and can provide an effective solution for industrial defect sample augmentation. Although the proposed method achieves favorable generation performance, it still has certain limitations. First, when the ROI prior is inaccurate, the generated anomaly may suffer from positional deviation, thereby affecting the spatial placement of the defect. Second, for defect categories with extremely large shape variations, relying only on reference anomalies for geometric constraints may limit generation diversity. In addition, when there is a large domain gap between the reference image and the target image, such as obvious differences in equipment, illumination conditions, or production batches, the cross-domain contextual consistency constraint may be insufficient to fully eliminate style shifts. Finally, this paper adopts existing ROI priors as inference inputs; therefore, its applicability still has room for improvement in scenarios where reliable region localization information is unavailable. In future work, automatic localization and adaptive region modeling can be further explored to develop a more robust training-free industrial defect generation framework.

Future work can focus on the following directions:

1. More robust reference-anomaly modeling strategies will be investigated. The current method still requires reference defect samples to provide anomalous appearance priors, and the quality, representativeness, and category coverage of the reference samples may affect the generation results. In future work, multi-reference sample fusion, reference-anomaly feature aggregation, or category-level anomaly prototype modeling will be considered to reduce the dependence on the quality of a single reference sample;
2. More efficient sampling strategies and lightweight guidance mechanisms will be explored. Since the multi-step reverse sampling process of diffusion models introduces certain inference overhead, future work will further investigate fewer-step sampling strategies, simplified computation of constraint terms, and lightweight frequency-domain/geometric guidance mechanisms. These improvements aim to enhance generation efficiency while maintaining the training-free property and generation quality, thereby better supporting large-scale defect sample augmentation;
3. The proposed method will be extended to more industrial products and more complex anomaly scenarios. Future work will further evaluate the applicability of the method on datasets with different materials, imaging conditions, and defect morphologies, with a focus on verifying its generalization ability across categories, materials, and scenarios.

### 5.2. Industrial Deployment

Compared with methods that require retraining the generative model, the proposed method does not rely on additional parameter updates, making it more suitable for rapid deployment and cross-scenario transfer in industrial sites. In practical industrial inspection systems, when the target product type, imaging condition, or defect pattern changes, conventional training-based generation methods usually require recollecting anomalous samples and fine-tuning the model. In contrast, the proposed method can directly use pretrained diffusion models to perform anomaly transfer and defect synthesis, making it more suitable for industrial environments with limited defect samples and fast production cycles.

In addition, the proposed method can improve downstream detection performance while maintaining a relatively low deployment cost, which has potential application value in high-reliability industrial scenarios such as pharmaceutical packaging, precision electronics, and transparent containers. Especially under conditions where low-frequency anomalies and long-tail anomalies are difficult to collect, the proposed method can serve as an anomalous sample augmentation module in industrial visual sensing systems, providing richer training data support for detection models.

## Figures and Tables

**Table 1:** Equipment configuration of the vial visual inspection system.

Category	Model	Manufacturer	Description
Industrial camera	Basler ALA1920-40GM	Basler AG (Ahrensburg, Germany)	1/1.2-inch CMOS sensor, resolution 1920 × 1200, frame rate 40 fps, GigE interface, supporting pylon 8.1.0.
Lens	Computar M1628-MPX	Computar (Tokyo, Japan)	Focal length 16 mm, maximum aperture F2.8, compatible with 2/3-inch sensors, supporting high-resolution imaging.
Light source	LDL2-19X4SW2(A)	CCS Inc. (Kyoto, Japan)	White light source, correlated color temperature 7000 K, dimensions W 28 × D 6.4 × H 13 mm, input voltage 24 V DC, maximum power consumption 1.3 W.

**Table 2:** Experimental parameter settings.

Parameter	Value
Batch size	24
Epoch	300
Optimizer	SGD
Learning rate	0.01
Workers	4
Image size	640×640
Training set	80%
Test set	10%
Validation set	10%

**Table 3:** Statistical analysis of vial defects.

Class	Avg (H:W)	Avg (Area)	Er (H:W)	Er (Area)
Bottle Expansion	1.0264	3811.16	1.1399	3.4886
Bottle Bottom	1.1171	2005.32	3.8872	3.9405
Cap Edge	1.4505	62361.12	2.5365	1.8596
Cake Surface	1.2762	18128.58	1.2100	1.5002
Cap Appearance	2.9680	41428.79	1.3342	1.5551
Bottle Appearance	0.7981	11376.21	1.7097	1.6978

**Table 4:** Diffusion model hyperparameter settings.

Source	Parameter	Value	Meaning
Overall weight	$\lambda_{m}$	0.44	Overall weight of the multi-domain consistency constraint module
Overall weight	$\lambda_{s}$	0.56	Overall weight of the geometric semantic constraint module
MDC	$\lambda_{fd}$	0.35	Weight of the frequency-domain consistency term
MDC	$\lambda_{ct}$	0.20	Weight of the content constraint term
MDC	$\lambda_{ctx}$	0.30	Weight of the local context consistency term
MDC	$\lambda_{stv}$	0.15	Weight of the style alignment term
SSC	η	0.60	Weight of the geometric structural constraint
SSC	ξ	0.40	Weight of the spatial semantic constraint
Geometric consistency	$\lambda_{grad}$	0.30	Weight of the gradient term
Geometric consistency	$\lambda_{geo}$	0.10	Weight of the geometric perturbation regularization term
Region anchoring	$\lambda_{ante}$	0.15	Weight of the region-anchored gating cost term
Region consistency	$\lambda_{roi}$	0.35	Weight of the region consistency loss

**Table 5:** Comparison of local IS and IC LPIPS of different anomaly generation methods on the MVTec AD dataset.

Category	Cut-Paste		DFMGAN		AnoDiff		AnoAny		TF-IDG		Ours	
	IS ↑	IC ↑	IS ↑	IC ↑	IS ↑	IC ↑	IS ↑	IC ↑	IS ↑	IC ↑	IS ↑	IC ↑
bottle	1.43	0.04	1.62	0.12	1.58	0.19	1.73	0.17	1.81	0.22	**1.94**	**0.26**
cable	1.74	0.25	1.96	0.25	2.13	0.41	2.06	0.41	2.19	0.44	**2.31**	**0.49**
capsule	1.23	0.05	1.59	0.11	1.59	0.21	2.16	0.23	2.24	0.26	**2.38**	**0.30**
carpet	**1.95**	0.11	1.23	0.13	1.16	0.24	1.10	0.34	1.29	**0.36**	1.26	0.34
grid	2.00	0.12	1.97	0.13	2.04	0.44	2.31	0.38	2.37	0.46	**2.52**	**0.50**
hazelnut	1.74	0.21	1.93	0.24	2.13	0.31	2.55	0.32	2.61	0.35	**2.74**	**0.39**
leather	1.47	0.14	2.06	0.17	1.94	0.41	2.26	0.41	**2.33**	**0.43**	2.19	0.42
metalnut	1.56	0.15	1.49	0.32	1.96	0.30	1.82	0.27	2.03	0.34	**2.16**	**0.38**
pill	1.49	0.11	1.63	0.16	1.61	0.26	2.91	0.30	2.98	0.33	**3.11**	**0.37**
screw	**1.39**	0.16	1.12	0.14	1.28	0.30	1.33	0.32	**1.39**	**0.35**	1.38	0.31
tile	1.83	0.20	2.39	0.22	2.54	0.55	2.66	0.53	2.73	0.58	**2.89**	**0.63**
toothbrush	1.30	0.08	1.82	0.18	1.68	0.21	1.64	0.22	1.87	0.25	**2.01**	**0.28**
transistor	1.39	0.15	1.64	0.25	1.57	0.34	1.66	0.28	1.72	0.37	**1.85**	**0.41**
wood	1.95	0.23	2.12	0.35	2.33	0.37	1.93	0.41	**2.41**	**0.44**	2.37	0.42
zipper	1.23	0.11	1.29	0.27	1.39	0.25	2.14	0.33	2.20	0.36	**2.34**	**0.40**
average	1.51	0.14	1.72	0.20	1.79	0.31	2.01	0.32	2.14	0.36	**2.23**	**0.39**

Note: ↑ indicates that higher values are better. Bold values indicate the best result in each row.

**Table 6:** Comparison of local IS and IC LPIPS of different anomaly generation methods on the vial dataset.

Category	Cut-Paste		DFMGAN		AnoDiff		AnoAny		TF-IDG		Ours	
	IS ↑	IC ↑	IS ↑	IC ↑	IS ↑	IC ↑	IS ↑	IC ↑	IS ↑	IC ↑	IS ↑	IC ↑
BE	1.62	0.09	1.88	0.16	2.01	0.24	2.17	0.27	2.28	0.31	**2.43**	**0.36**
BB	1.58	0.08	1.81	0.15	1.95	0.22	2.11	0.25	**2.24**	**0.30**	2.12	0.28
CE	1.71	0.12	1.94	0.18	2.08	0.26	2.26	0.29	2.39	0.33	**2.56**	**0.38**
CS	1.49	0.07	1.73	0.13	1.88	0.19	2.02	0.23	**2.16**	**0.27**	2.09	0.22
CA	1.55	0.10	1.79	0.16	1.93	0.23	2.12	0.26	2.24	0.30	**2.40**	**0.35**
BA	1.60	0.09	1.84	0.15	1.98	0.22	2.14	0.24	2.27	0.29	**2.45**	**0.34**
average	1.59	0.09	1.83	0.15	1.97	0.22	2.13	0.25	2.26	0.30	**2.34**	**0.32**

Note: ↑ indicates that higher values are better. Bold values indicate the best result in each row.

**Table 7:** Comparison of downstream detection results of YOLOv11 using samples generated by different augmentation methods on the MVTec AD dataset (I).

Model	Category	Without Generative Model			DFMGAN			AnoDiff		
		P	R	AP	P	R	AP	P	R	AP
YOLOv11	bottle	88.6	86.4	89.1	89.9	88.0	90.7	91.0	89.3	91.9
	cable	82.7	78.9	81.3	84.7	81.7	83.8	85.8	83.0	85.0
	capsule	89.4	87.2	89.8	90.7	88.8	91.4	91.8	90.1	92.6
	carpet	80.5	76.8	79.1	82.5	79.6	81.6	83.6	80.9	82.8
	grid	78.6	74.9	77.4	80.6	77.7	79.9	81.7	79.0	81.1
	hazelnut	90.2	88.4	91.1	91.5	90.0	92.7	92.6	91.3	93.9
	leather	84.8	81.3	84.2	86.8	84.1	86.7	87.9	85.4	87.9
	metalnut	91.0	89.2	92.0	92.3	90.8	93.6	93.4	92.1	94.8
	pill	88.7	85.6	88.4	90.0	87.2	90.0	91.1	88.5	91.2
	screw	81.3	77.5	80.2	83.3	80.3	82.7	84.4	81.6	83.9
	tile	83.6	79.1	82.4	85.6	81.9	84.9	86.7	83.2	86.1
	toothbrush	87.5	84.7	88.0	88.8	86.3	89.6	89.9	87.6	90.8
	transistor	79.4	73.8	78.2	81.4	76.6	80.7	82.5	77.9	81.9
	wood	82.1	77.9	81.0	84.1	80.7	83.5	85.2	82.0	84.7
	zipper	80.7	76.4	79.3	82.7	79.2	81.8	83.8	80.5	83.0
	average	85.8	81.8	83.8	87.3	83.2	85.2	88.1	84.0	86.0

**Table 8:** Comparison of downstream detection results of YOLOv11 using samples generated by different augmentation methods on the MVTec AD dataset (II).

Model	Category	AnoAny			TF-IDG			Ours		
		P	R	AP	P	R	AP	P	R	AP
YOLOv11	bottle	91.8	90.1	92.8	92.6	91.3	93.9	93.2	92.0	94.7
	cable	86.6	83.8	85.9	87.4	85.0	87.0	88.0	85.7	87.8
	capsule	92.6	90.9	93.5	93.4	92.1	94.6	94.0	92.8	95.4
	carpet	84.4	81.7	83.7	85.2	82.9	84.8	85.0	82.6	84.6
	grid	82.5	79.8	82.0	83.3	81.0	83.1	83.9	81.7	83.9
	hazelnut	93.4	92.1	94.8	94.2	93.3	95.9	94.8	94.0	96.7
	leather	88.7	86.2	88.8	89.5	87.4	89.9	89.1	87.1	89.7
	metalnut	94.2	92.9	95.7	95.0	94.1	96.8	95.6	94.8	97.6
	pill	91.9	89.3	92.1	92.7	90.5	93.2	93.3	91.2	94.0
	screw	85.2	82.4	84.8	86.0	83.6	85.9	86.6	82.9	85.7
	tile	87.5	84.0	87.0	88.3	85.2	88.1	87.9	85.9	88.9
	toothbrush	90.7	88.4	91.7	91.5	89.6	92.8	92.1	90.3	93.6
	transistor	83.3	78.7	82.8	84.1	79.9	83.9	84.7	80.6	84.7
	wood	86.0	82.8	85.6	86.8	84.0	86.7	86.4	83.7	86.5
	zipper	84.6	81.3	83.9	85.4	82.5	85.0	86.0	83.2	85.8
	average	89.4	85.3	89.3	90.6	86.5	88.5	91.8	87.6	89.6

**Table 9:** Comparison of downstream detection results of YOLOv11 using samples generated by different augmentation methods on the vial dataset (I).

Model	Category	Without Generative Model			DFMGAN			AnoDiff		
		P	R	AP	P	R	AP	P	R	AP
YOLOv11	BE	95.0	98.8	98.1	96.2	99.1	98.5	96.8	99.3	98.8
	BB	95.2	95.6	96.0	96.0	96.8	96.8	96.6	97.5	97.5
	CE	91.3	68.0	85.6	92.5	74.8	88.7	93.4	79.2	90.6
	CS	99.0	100.0	99.5	99.1	100.0	99.5	99.3	100.0	99.6
	CA	99.4	100.0	99.5	99.4	100.0	99.6	99.5	100.0	99.6
	BA	92.2	93.8	96.0	93.4	95.0	96.8	94.1	95.9	97.3
	average	94.3	94.8	95.6	94.9	97.3	97.0	96.1	94.3	97.0

**Table 10:** Comparison of downstream detection results of YOLOv11 using samples generated by different augmentation methods on the vial dataset (II).

Model	Category	AnoAny			TF-IDG			Ours		
		P	R	AP	P	R	AP	P	R	AP
YOLOv11	BE	97.4	99.4	99.0	98.1	99.5	99.3	97.9	99.6	99.4
	BB	97.3	98.1	98.2	98.0	98.5	98.8	97.8	98.3	98.4
	CE	94.1	82.6	91.9	95.0	86.7	93.8	95.6	89.8	94.9
	CS	99.5	100.0	99.6	99.7	100.0	99.7	99.6	99.9	99.5
	CA	99.6	100.0	99.7	99.8	100.0	99.7	99.7	100.0	99.8
	BA	95.0	96.6	97.9	96.1	97.4	98.6	96.4	97.8	98.9
	average	96.9	95.4	98.0	96.9	98.1	98.0	97.4	97.4	98.8

**Table 11:** Stability analysis of YOLOv11 downstream detection results over three repeated runs.

Dataset	Method	mAP@50
MVTec AD	TF-IDG	88.5 ± 0.2
MVTec AD	Ours	89.6 ± 0.1
Self-constructed vial	TF-IDG	98.0 ± 0.1
Self-constructed vial	Ours	98.8 ± 0.1

**Table 12:** Supplementary downstream detection results using YOLOv26 on the MVTec AD dataset.

Class	P	R	mAP@50
Bottle	94.2	93.5	95.8
Cable	89.5	87.2	88.9
Capsule	95.5	94.0	96.2
Carpet	86.5	84.2	86.2
Grid	85.2	83.1	85.4
Hazelnut	96.0	95.2	97.8
Leather	91.0	88.8	91.5
Metal Nut	96.8	96.0	98.5
Pill	94.5	92.5	95.2
Screw	88.0	85.0	87.2
Tile	89.8	87.5	90.5
Toothbrush	93.5	91.8	94.8
Transistor	86.2	82.5	86.4
Wood	88.2	85.8	88.0
Zipper	87.5	84.8	87.3
Average	93.5	89.5	94.5

**Table 13:** Supplementary downstream detection results using YOLOv26 on the self-constructed vial defect dataset.

Class	P	R	mAP@50
BE	98.6	99.1	99.5
BB	97.5	94.2	97.9
CE	96.8	91.5	95.3
CS	99.7	100.0	99.8
CA	97.9	97.3	99.6
BA	99.6	99.2	99.3
Average	98.1	96.3	98.7

**Table 14:** Comparison of ablation experimental results on the vial dataset.

Method	FDG	CAF	ESC	SRA	Local IS	IC LPIPS	YOLOv11 mAP (%)
TF-IDG	×	×	×	×	2.26	0.30	98.0
TF-IDG+FDG	✓	×	×	×	2.29	0.30	98.3
TF-IDG+CAF	×	✓	×	×	2.28	0.31	98.2
TF-IDG+FDG+CAF	✓	✓	×	×	2.31	0.31	98.5
TF-IDG+ESC+SRA	×	×	✓	✓	2.30	0.31	98.4
TF-IDG+FDG+CAF+ESC	✓	✓	✓	×	2.33	0.32	98.7
Proposed method	✓	✓	✓	✓	2.34	0.32	98.8

Note: ✓ indicates that the corresponding module is used, whereas × indicates that the corresponding module is not used.

**Table 15:** Comparison of efficiency and resource consumption of different methods in high-resolution generation tasks on the vial dataset.

Method	Resolution	Sampling Steps	Peak GPU Memory Usage (GB)	Average Generation Time (s/Image)	Intermediate Attention Cache Size (GB)
Original TF-IDG	1024×1024	50	10.7	16.8	2.41
Ours	1024×1024	50	8.9	15.2	1.63

## Data Availability

Access to the dataset by contacting the corresponding author.
